# Effects of human-made resource hotspots on seasonal spatial strategies by a desert pitviper

**DOI:** 10.1038/s41598-019-52957-1

**Published:** 2019-11-13

**Authors:** Dominic L. DeSantis, Amy E. Wagler, Vicente Mata-Silva, Jerry D. Johnson

**Affiliations:** 1 Department of Biological Sciences, University of Texas at El Paso, El Paso, TX 79968 USA; 2 Department of Mathematical Sciences, University of Texas at El Paso, El Paso, TX 79968 USA

**Keywords:** Behavioural ecology, Herpetology

## Abstract

Habitat heterogeneity and local resource distribution play key roles in animal search patterns. Optimal strategies are often considered for foraging organisms, but many of the same predictions are applicable to mate searching. We quantified movement and space use by a pitviper to test whether Native Habitats (NH) and human-made Resource Hotspots (RH) facilitate alternative seasonal spatial strategies as a result of critical resources, including potential mating partners, being widely dispersed in NH and clustered in RH. Independent of habitat category, seasonal patterns resembled an intermediate mating system with elements of prolonged male mate-searching and female-defense. However, individuals using primarily NH or RH exhibited alternative strategies. NH rattlesnakes displayed greater movement and larger home ranges than RH rattlesnakes across behavioral seasons. NH males increased movement distances and home ranges during the mating season, while RH males displayed minimal or no seasonal shifts. NH females also elevated movement distances during the mating season, while RH females showed no significant seasonal differences. Despite contrasting spatial patterns, mating success and female-defense effort were not significantly affected by habitat category. This unique study system highlights the potential for interactions among sexual selection, habitat heterogeneity, and behavioral plasticity to facilitate divergent search tactics within populations.

## Introduction

Animals move through environments, in part, to locate resources needed for survival, such as food, water, and shelter. Patterns of animal movement and space use are, therefore, shaped in response to spatiotemporal variation in the distribution of these critical resources. Movement is also costly, as it increases energy usage and exposure risks, and ecologists have extensive history of testing for optimization of behavior in the context of this trade-off^1–3^. However, numerous endogenous and exogenous factors produce variation in what might be considered optimal strategies among individuals. Habitat heterogeneity is one such factor, as variation in habitat structure directly affects the spatiotemporal arrangement of ecological resources in the environment^4–6^. Consequently, local resource distributions drive the spatial dispersion of individuals across a landscape and their respective movement and space use patterns.

Animal foraging is most often studied to identify the role of habitat heterogeneity in movement or search strategies^4,7,8^. However, the general predictions of optimality theory are also applicable to mate-searching behavior, as potential mating partners represent discrete resources located through space and time^9^. Sexual selection has frequently resulted in male movement being an effective mechanism for locating potential mates^10^. For vertebrate mating systems that lack male parental care, female reproductive success depends primarily on access to ecological resources. The distribution of limiting ecological resources, therefore, drives female spatial dispersion and search strategies by reproductive males (during mating periods) are shaped in response^11–13^. Accordingly, variation in habitat quality across a heterogeneous landscape can play an important role in movement and space use strategies in sex and season-specific contexts^14^. For example, shorter movements and reduced space use should be favored for males when females are spatially clustered, as the costs of moving (i.e., energy expenditure and predation risk) are minimized without sacrificing the benefits due to increased encounter rates^1–3^. In landscapes where female distribution might vary across different habitat patches, males are expected to adopt search strategies that reflect local conditions^15^. In modified environments containing novel patches of resource-rich or deficient habitat, this behavioral plasticity can be a critical adaptive mechanism.

Although historically understudied in behavioral ecology^16,17^, snakes, and pitvipers, specifically, are intriguing model organisms for field studies of sex and season-specific movement and space use strategies^18–20^. As primarily solitary organisms, there is relative ease in identifying reproductive behavior, and male movement is the primary mate location mechanism^21^. Considerable variation in reproductive strategies inferred from spatial patterns has been reported, often in response to the timing of the mating season and spatial distribution of females^13,18^. This plasticity combined with strong seasonality in motivational states make snakes ideal for investigating relationships between behavioral season, environmental conditions, and search patterns^16,22,23^. We aimed to explore the drivers of spatial strategies by a cryptic pitviper (western diamond-backed rattlesnakes, *Crotalus atrox*) in a unique study system within the arid northern Chihuahuan Desert. Past construction of earthen tanks (ephemeral ponds) on our study site has created potential resource hotspots (RH) relative to native habitats (NH). We exploit this human-made variation in habitat structure and quality to test whether differences in local resource distribution drive divergent movement and space use strategies across behavioral seasons by proximate rattlesnakes. We expected seasonal patterns to reflect a male search-based polygynandrous mating system^24^. Males were predicted to increase movement and space use during the mating season in mate-searching efforts, and females were predicted to show no seasonal differences. As in most taxa, female snakes have historically been characterized as passive players in mate location, and often display no seasonal differences in movement behavior^25,26^. However, cryptic female choice, benefits of multiple mating (sperm competition and multiple paternity), and pheromone signaling implicate a potentially larger role than previously considered^12,17,23^. When accounting for habitat category, we expected differences in local resource abundance to facilitate divergent spatial patterns. Independent of season, RH rattlesnakes were predicted to reduce movement and space use relative to NH counterparts. In response to the local distribution of limiting ecological resources, RH females were expected to be spatially clustered during the mating season while NH females were expected to be more widely dispersed. We, therefore, predicted that RH males would display minimal or no seasonal differences in movement and space use compared to NH males, which were expected to increase movement during the mating season in prolonged mate-searching efforts. Finally, we integrated spatial data with observations of mating behavior to further evaluate the effects of habitat heterogeneity on reproductive strategies.

## Methods

### Study site and species

All methods were carried out in accordance with relevant guidelines and regulations, and all research protocols were approved for the entire study period by the University of Texas at El Paso Animal Care and Use Committee (Protocol A-201405-1) and Texas Parks and Wildlife (Scientific Permit Number SPR–0290–019). Field data collection occurred at the 16,000 Ha Indio Mountains Research Station (IMRS; centered on 30.75°N, 105.00°W), a University of Texas at El Paso controlled property within the northern Chihuahuan Desert in Hudspeth County, Texas. Detailed descriptions of IMRS can be found elsewhere^27,28^, as we provide only a summary of relevant information. Mean annual precipitation is 235 mm, with most rainfall (≈70%) occurring during late summer (July–September). During the active season for *C. atrox* (April–October) within the study period (June 2015–August 2018), mean daily temperature was 25.5 °C with a mean daily maximum and minimum of 32.3 °C and 18.4 °C, respectively.

Western diamond-backed rattlesnakes (*C. atrox*) are large bodied pitvipers (Serpentes: Viperidae) distributed throughout the southwestern United States into northern and central Mexico^29^. As one of the most widespread and abundant rattlesnakes across its geographic range, *C. atrox* has been the subject of numerous ecological investigations^30–33^, although a relative paucity of literature exists for the species in the Chihuahuan Desert. Like most pitvipers (Crotalinae), *C. atrox* are primarily sit-and-wait (i.e., ambush) predators, with rodents and lagomorphs comprising the vast majority of their diet^34,35^. Considerable geographic variation in average adult body size is observed across their range^36^, but male-biased sexual size dimorphism is always maintained. Phenological studies of *C. atrox* behavior have shown that they engage in two discrete annual mating seasons, initially during late summer and again in spring after egress from winter shelters^37,38^. However, over 30 years of field work on IMRS, reproductive behavior has only been observed from late summer through fall. We therefore considered the annual mating season for *C. atrox* on IMRS to be restricted to a single three-month period from August through October. The mating system for *C. atrox* in Arizona was recently described as “attendant polygynandry,” as males and females often mate with multiple partners within a mating period, males act as the mate-searching sex, and extended female attendance and male-male combat (i.e., female-defense) were observed^17^. The relatively small scale of space use and high densities of *C. atrox* on IMRS made it an ideal model organism to test for the effects of historical human manipulation of habitat on spatial and reproductive strategies. Large earthen embankments were built in several arroyos on IMRS to create ephemeral ponds (i.e., earthen tanks) when the property supported livestock. Only a handful of tanks still function as ephemeral water bodies and they are widely dispersed across the property. Outside of these features, surface water is rarely available on IMRS with the exception of a single permanent spring.

### Movement and space use

Radio telemetry was used to collect movement and space use data from 35 *C. atrox* (18 males, 17 females) between June 2015 and August 2018. Three additional females were radiotracked during the study period but were not included in analyses due to being confirmed as gravid during telemetry monitoring. Two additional males were lost to unknown predators shortly after the onset of monitoring and also did not contribute data to analyses. Radio transmitters (Holohil Systems Ltd., Models SB-2T and SI-2T) were internally implanted in rattlesnakes^39^ and comprised ≤5% of each individual’s body mass at the time of the procedure. Rattlesnakes were released at the site of capture one to three days following implantation, and relocations occurred every two to three days during the active season (April–October) and biweekly during the inactive season (November–March). Locations were recorded in Universal Transverse Mercator (UTM) with a hand-held global positioning system (Garmin: Oregon 400t). Detailed behavioral observations were made during each relocation and the used habitat classification was recorded. For analysis of reproductive behavior, the number of mating partners observed for each individual (i.e., courtship and coitus observed) along with the minimum number of days spent in attendance per partner were recorded during the mating season.

Movement was quantified using a series of metrics, including Meters Per Day (MPD), calculated as the straight-line distance between successive relocation points for an individual, Distance Per Movement (DPM), calculated as the mean straight-line distance between relocation points that were ≥5 m apart, and Minimum Movement Frequency (MMF), calculated as the number of movements (≥5 m) made by an individual out of *N* relocations in a specifically defined time period^40^. Directionality of movements was calculated by obtaining the bearing angle between successive locations that constituted a movement by an individual. Bearings were grouped by sex and season to calculate circular variance as a proxy for directionality^40^. Circular variance is a number between 0 and 1, with values closer to 0 indicating more linear movements. Annual movement measures were calculated and condensed into non-mating and mating seasons for analysis.

A fundamental spatial expression of an animals’ series of movement steps has long been recognized as the home range^41^. This macro-scale estimation of space use allows interpretation of how individual characteristics and the external environment combine to drive spatial strategies^42^. For this study, we selected a suite of home range estimators commonly presented in studies of snake spatial ecology^43^. Home range sizes were derived using 100% Minimum Convex Polygons (MCP) and 95% fixed-kernel Utilization Distributions (UD) with the plug-in bandwidth matrix, and core use areas were estimated with 50% UDs also using the plug-in bandwidth matrix^20,44^. We selected the plug-in bandwidth matrix for UD home ranges because it was demonstrated as being robust to variation in sampling rate and duration for a congener of *C. atrox*^44^. Seasonal home ranges were calculated only for individuals tracked across every month of a non-mating (April–July) or mating season (August–October). Movement metrics and home range sizes were calculated in R using the *adehabitat*, *adehabitatHR*, *adehabitatLT*, and *circular* packages^45,46^.

### Habitat heterogeneity

**Figure 1:**
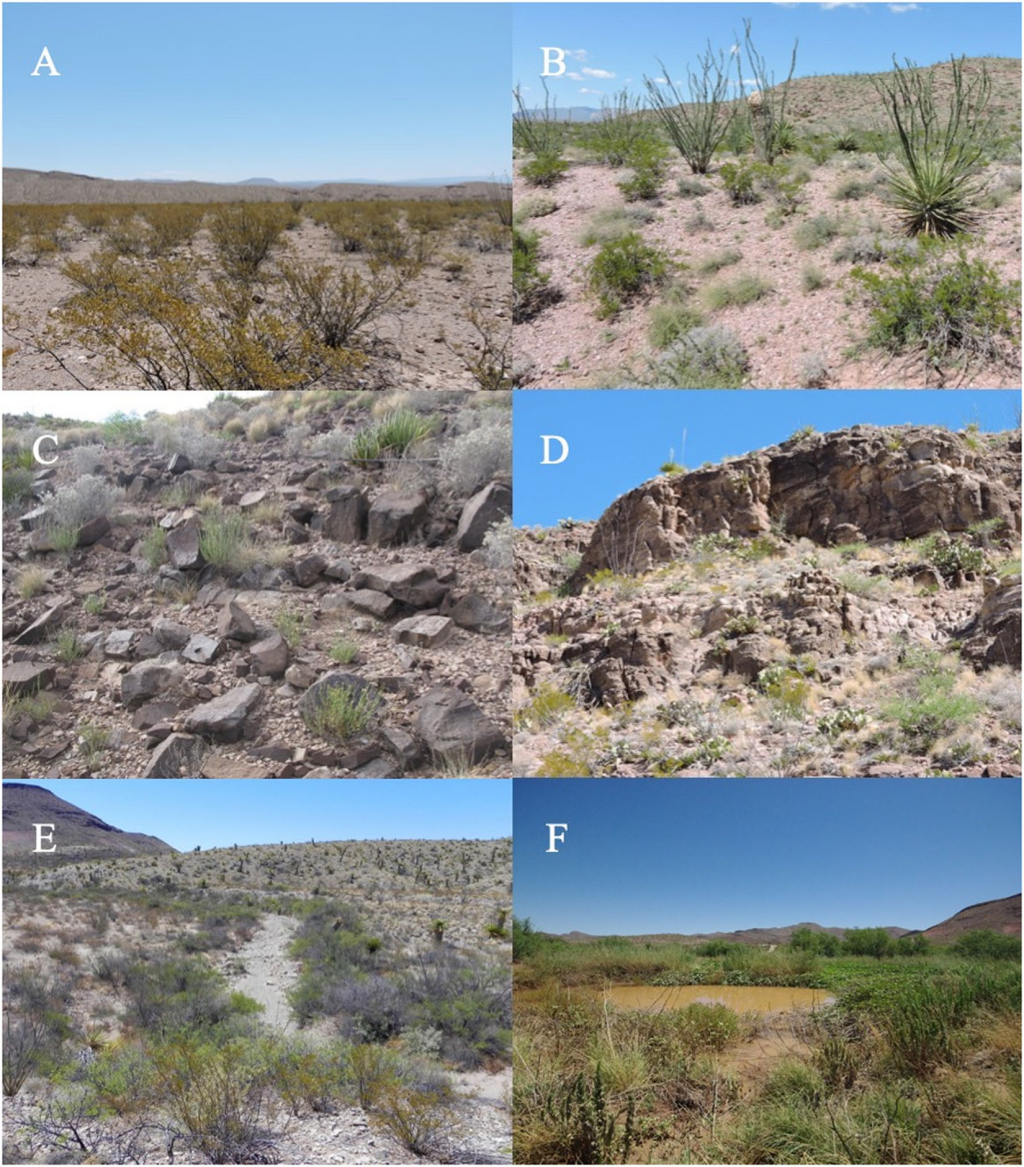
Representative images of habitat classifications. (**A**) Alluvial Flat, (**B**) Alluvial Slope, (**C**) Alluvial Rocky Slope, (**D**) Rocky Slope, (**E**) Arroyo, and (**F**) Earthen Tank. *Native Habitat* (NH) = (**A**–**E**); *Resource Hotspot* (RH) = F. Photos taken by VMS (**A**–**E**) and DLD (**F**) at Indio Mountains Research Station, Hudspeth County, Texas.

Rattlesnakes were grouped into two habitat categories for analysis: “resource hotspot” (RH) and “native habitat” (NH). Placement into each category was determined using the proportion of relocations in each habitat classification by individuals. Individuals that used earthen tank habitat more frequently than any other one habitat class during a specified time period (i.e., year or season) were placed into the RH category. Individuals that did not meet this criterion were placed into the NH category. Habitat classifications included: alluvial flat, alluvial slope, alluvial rocky slope, rocky slope, arroyo, and earthen tank (Fig. 1).

To test the prediction that earthen tank habitats represented relative resource hotspots for *C. atrox* on IMRS, prey abundance was estimated in each habitat class via rodent and lagomorph fecal pellet counts. Pellet counts are a validated and non-invasive method for estimating local small mammal abundance^47–49^. Counts were conducted within a m^2^ quadrat where all visible rodent and lagomorph pellets were tallied. Ten quadrat counts were conducted at 10 m intervals along a 100 m transect through each habitat class. Transects ran at a random bearing from a UTM coordinate located at a random distance (10–100 m) and bearing from a relocation point of a radiotracked rattlesnake located within the selected habitat class^50,51^. Pellet counts were conducted twice annually (once in April and once in August) in each habitat class during the two full years of radio telemetry data collection (2016 and 2017).

To further explore potential relationships between habitat category and the spatial patterns of proximate rattlesnakes, we calculated an index of dispersion (*I*) to quantify the relative dispersion of radiotracked rattlesnakes over time. *I* was calculated as the variance-to-mean ratio of the distance to the nearest neighbor for individuals at weekly intervals (i.e., every other relocation)^40^. When *I* is equal to one, this indicates a random distribution, while values less than one and greater than one indicate a dispersed and clumped distribution pattern, respectively^52^. This measure is not intended to provide absolute estimates of the spatial distribution of individuals in this population (which would require tracking nearly every individual in a given area)^40^, but instead may serve as a proxy for how the relative dispersion of *C. atrox* on IMRS might vary over time relative to habitat category and behavioral season.

### Statistical analysis

Generalized Linear Mixed-effects Models (GLMMs) were employed to test for the effects of sex, behavioral season, and habitat category on movement and space use patterns^53^. Response variables (movement metrics and home range estimations) were modeled with separate GLMMs using the logit link. Year and individual rattlesnake within year were modeled as random effects to control for non-independence of data across time. Fixed effects included sex (male, female), behavioral season (non-mating, mating), and habitat category (RH, NH). GLMMs were also used to evaluate how reproductive behavior was associated with sex, body size (Snout-to-Vent-Length; SVL), movement measures (MPD, DPM, MMF, Directionality), space use estimates (100% MCP, 95% UD, 50% UD), and habitat category (RH, NH). The two response variables (number of mating partners, attendance-days-per-partner) were modeled using separate GLMMs and rattlesnake ID was included in the model as a random effect. In an exploratory analysis, appropriate transformations for each response variable were determined and employed in the model. This resulted in the following set of response variable transformations: MPD (log base 10), DPM (log base 10), MMF (Logit), Directionality (Logit), 100% MCP (Log Base 10), 95% UD (Log Base 10), 50% UD (Log Base 10). For the Directionality and MMF response variables, beta regression models were employed, but the models using the logit link with random effects for ID provided a superior fit. Intra-class correlation coefficient (ICC) and variance were used in comparisons of model parsimony, marginal and conditional pseudo-*R*^2^ measures were used to evaluate model fit, and 95% confidence intervals and *P*-values illustrate the effects of factor levels. Marginal pseudo-*R*^2^ describes the proportion of model variance explained by fixed effects only, while conditional pseudo-*R*^2^ describes the proportion of variance explained by both fixed and random effects. Pairwise least-squared mean interval estimates were computed using the general linear hypothesis test and employing multiplicity adjustments^54^. A nonparametric ANOVA (Kruskal-Wallis) was used to test for differences in fecal pellet density among the four habitat classifications on IMRS, and Tukey’s pairwise comparisons were used to evaluate differences between classes. Goodness of fit tests were used to determine if the mean dispersion (*I*) of males and females grouped within habitat categories (NH and RH) differed from random. For all analyses, *a* was set at 0.05.

## Results

**Figure 2:**
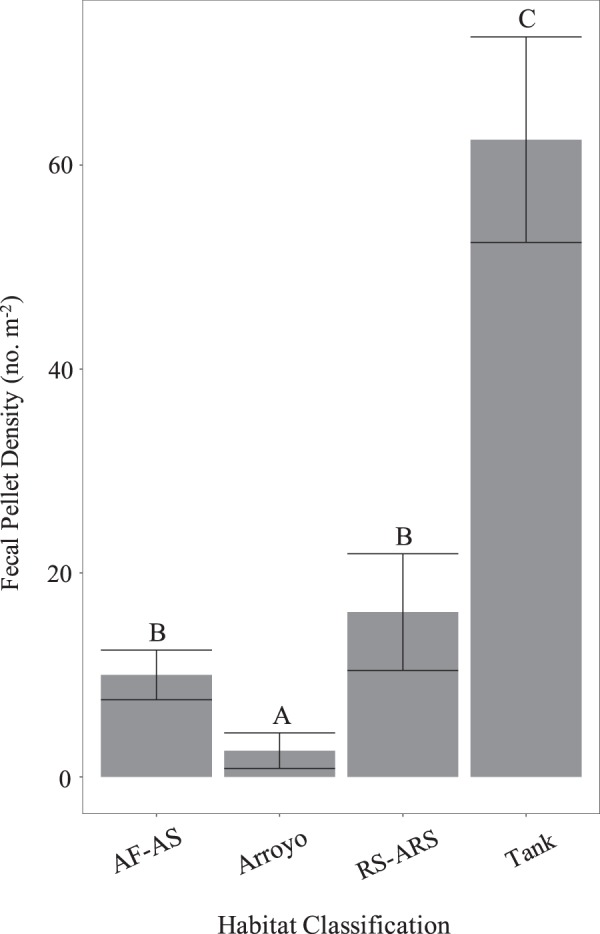
Mean (±s.e.m.) rodent and lagomorph fecal pellet density (no. m^−2^) in habitat classifications on IMRS. Data were pooled across two sampling years (2016–2017). Classes include Alluvial Flat and Alluvial Slope (AF-AS), Arroyo, Rocky Slope and Alluvial Rocky Slope (RS-ARS), and Earthen Tank (Tank). The AF-AS and RS-ARS classes were paired for analysis because single sampling transects often intersected both habitats due to their interconnectedness on IMRS. Letters above bars denote differences derived from post-hoc Tukey’s HSD pairwise mean comparisons (α = 0.05).

**Figure 3:**
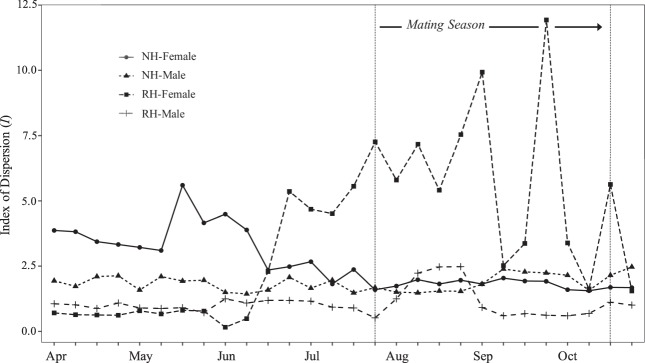
Seasonal variation in Index of Dispersion (*I*) for radiotracked *Crotalus atrox* from 2015–2018. Lines correspond to RH Female (*N* = 8), NH Female (*N* = 9), RH Male (*N* = 9), and NH Male (*N* = 9) groups. Points along individual lines are weekly variance-to-mean ratios, with values greater than one indicating a “clustered” spatial distirbution and those less than one indicating a “dispersed” spatial distirbution. The distirbutions of all groups were significantly different from random (i.e., Poisson): RH Female (*Χ*^2^ [28, *N* = 8] = 1.73e^20^, *P* < 0.01), NH Female (*Χ*^2^ [28, *N* = 9] = 1.57e^20^, *P* < 0.01), RH Male (*Χ*^2^ [28, *N* = 9] = 1.40e^20^_,_ *P* < 0.01), NH Male (*Χ* ^2^ [28, *N* = 9] = 4.73e^20^, *P* < 0.01). Note the increase in spatial clustering of RH females during the mating season, while all other radiotracked groups exhibit comparatively little seasonal variation in *I*.

Individual rattlesnakes were radiotracked for durations ranging from 70–918 days (mean ± s.d. = 380 ± 188) between June 2015 and August 2018 for a cumulative total of 2577 telemetry relocations (mean ± s.d. = 74 ± 44). Male (*N* = 18) and female (*N* = 17) annual movement and space use measures were calculated (Table 1) and condensed into behavioral seasons (non-mating, mating) and habitat categories (RH, NH) for analyses. In total, 25 RH snake-season data points (14 non-mating, 11 mating) and 46 NH snake-season data points (24 non-mating, 22 mating) were accumulated. The 25 RH snake-seasons ranged from 0.32–0.90 in proportion of observations in earthen tanks (mean ± s.d. = 0.55 ± 0.18), while the 46 NH snake-seasons ranged from 0.0–0.18 (mean ± s.d. = 0.02 ± 0.05). Combined (rodent and lagomorph) fecal pellet density was highest in earthen tank habitat, followed by alluvial rocky slopes and rocky slopes, alluvial slopes and flats, and arroyos (Kruskal-Wallis ANOVA: H_3_ = 83.27; *P* < 0.01; Fig. 2). The distributions of *I* were statistically different from random for radiotracked snakes (RH Female, NH Female, RH Male, NH Male; see Fig. 3 caption). All groups were generally “clustered” throughout the year with RH females exhibiting the greatest seasonal variation in *I* (Fig. 3). GLMM results for movement, space use, and reproductive behavior are presented below (Tables 2, 3). *F-*statistics and *P*-values are provided in-text, coefficients and 95% confidence intervals can be referenced to further evaluate the effects (Tables 4, 5).

**Table 1:** Mean annual movement and space use measures (±s.e.m.) for male (*N* = 18) and female (*N* = 17) *Crotalus atrox* radiotracked between June 2015 and August 2018.

	MPD	DPM	MMF	Directionality	100% MCP	95% UD	50% UD
Males	51.06 ± 3.9	160.33 ± 10.5	0.76 ± 0.03	0.79 ± 0.03	22.68 ± 2.76	42.55 ± 6.12	7.35 ± 1.46
Females	25.89 ± 2.2	84.07 ± 5.7	0.69 ± 0.03	0.88 ± 0.01	4.31 ± 0.65	9.88 ± 1.78	1.07 ± 0.52

Movement measures include Meters Per Day (MPD, m), Distance Per Movement (DPM, m), Minimum Movement Frequent (MMF), and circular variance (Directionality). For Directionality, values closer to 0 indicate more linear movements. Space use measures include 100% Minimum Convex Polygon (100% MCP, Ha), 95% Utilization Distribution (95% UD, Ha), and 50% Utilization Distribution (50% UD/core use area, Ha).

**Table 2:** Marginal and conditional-*R*^2^, Intraclass Correlation Coefficient (ICC-Year, ICC-ID), and Variance for individual movement and space use GLMMs.

Model	Marignal-*R*^2^	Conditional-*R*^2^	ICC-Year	ICC-ID	Variance
MPD	0.33	0.68	0.12	0.33	0.01
DPM	0.56	0.89	0.54	0.21	0.01
MMF	0.30	0.50	0.04	0.25	1.0e-4
Directionality	0.21	0.56	0.45	1.0e-10	0.39
100% MCP	0.56	0.74	0.12	0.29	0.02
95% UD	0.50	0.76	0.52	5.5e-10	0.07
50% UD	0.42	0.89	0.45	0.35	0.25

**Table 3:** Marginal and conditional-*R*^2^, Intraclass Correlation Coefficient (ICC-Year, ICC-ID), and Variance for individual reproductive behavior GLMMs.

Model	Marignal-*R*^2^	Conditional-*R*^2^	ICC-ID	Variance
Mating Partners	0.20	0.70	0.70	0.78
Female Attendance	0.51	0.64	0.69	0.89

**Table 4:** Coefficients, 95% confidence intervals, and *P*-values for movement and space use model parameters.

Model *Parameter*	Coefficient	95% Confidence Interval		*P*
Meters Per Day		*Lower*	*Upper*	
*Sex* (*Female*)	−0.36	−0.58	−0.15	<0.01
*Season* (*NM*)	−0.28	−0.46	−0.10	<0.01
*Habitat* (*RH*)	−0.07	−0.30	0.17	0.20
Sex*Season	0.21	−0.03	0.45	<0.01
Sex*Habitat	−0.04	−0.40	0.30	0.97
Season*Habitat	−0.08	−0.39	0.25	0.77
Sex*Season*Habitat	0.09	−0.37	0.54	0.70
**Distance Per Movement**				
*Sex* (*Female*)	−0.22	−0.34	−0.09	<0.01
*Season* (*NM*)	−0.10	−0.18	−0.02	0.10
*Habitat* (*RH*)	−0.15	−0.28	−0.02	<0.01
Sex*Season	−0.04	−0.18	0.07	0.61
Sex*Habitat	−0.17	−0.37	0.02	0.30
Season*Habitat	0.08	−0.08	0.25	0.01
Sex*Season*Habitat	0.15	−0.07	0.36	0.22
**Minimum Movement Frequency**				
*Sex* (*Female*)	−0.17	−0.34	−0.01	0.19
*Season* (*NM*)	−0.35	−0.50	−0.19	<0.01
*Habitat* (*RH*)	0.09	−0.10	0.28	0.18
Sex*Season	0.34	0.11	0.55	0.15
Sex*Habitat	0.03	−0.27	0.30	0.19
Season*Habitat	0.16	−0.14	0.44	0.71
Sex*Season*Habitat	−0.39	−0.78	0.04	0.05
**Directionality**				
*Sex* (*Female*)	0.58	−0.15	1.32	0.13
*Season* (*NM*)	−0.46	−1.05	0.16	0.56
*Habitat* (*RH*)	0.61	−0.18	1.40	0.15
Sex*Season	0.74	−0.15	1.63	0.23
Sex*Habitat	−0.90	−2.04	0.25	0.03
Season*Habitat	0.65	−0.58	1.88	0.33
Sex*Season*Habitat	−0.47	−2.07	1.15	0.58
**100% Minimum Convex Polygon**				
*Sex* (*Female*)	−0.72	−1.02	−0.41	<0.01
*Season* (*NM*)	−0.22	−0.49	0.03	<0.01
*Habitat* (*RH*)	−0.30	−0.63	0.03	<0.01
Sex*Season	−0.04	−0.40	0.34	0.91
Sex*Habitat	0.14	−0.34	0.62	0.45
Season*Habitat	−0.21	−0.67	0.29	0.25
Sex*Season*Habitat	0.04	−0.62	0.65	0.90
**95% Utilization Distribution**				
*Sex* (*Female*)	−0.63	−0.92	−0.31	<0.01
*Season* (*NM*)	−0.37	−0.60	−0.12	0.03
*Habitat* (*RH*)	−0.35	−0.66	−0.03	<0.01
Sex*Season	0.24	−0.12	0.63	0.28
Sex*Habitat	−0.07	−0.63	0.48	0.62
Season*Habitat	0.07	−0.39	0.53	0.86
Sex*Season*Habitat	−0.07	−0.99	0.74	0.85
**50% Utilization Distribution**				
*Sex* (*Female*)	−0.89	−1.47	−0.26	0.01
*Season* (*NM*)	−0.55	−0.89	−0.19	<0.01
*Habitat* (*RH*)	−0.69	−1.21	−0.12	<0.01
Sex*Season	0.05	−0.47	0.56	0.39
Sex*Habitat	0.19	−0.95	1.09	0.40
Season*Habitat	−0.53	−1.23	0.14	0.27
Sex*Season*Habitat	0.41	−0.75	1.65	0.49

Reference levels for parameters are in parentheses (Female, NM = Non-mating Season, RH = Resource Hotspot), and coefficients can be used to interpret the direction of individual effects.

**Table 5:** Coefficients, 95% confidence intervals, and *P*-values for reproductive behavior model parameters.

Model *Parameter*	Coefficient	95% Confidence Interval		*P*
Number of Mating Partners		*Lower*	*Upper*	
*Sex* (*Male*)	−1.55	−0.78	0.09	0.15
*Habitat* (*RH*)	0.91	−0.11	0.32	0.38
*SVL*	1.43	−0.82	5.32	0.17
*MPD*	−0.32	−0.63	0.45	0.75
*DPM*	0.66	−0.45	0.91	0.52
*MMF*	0.01	−0.16	0.16	0.99
*Directionality*	−0.08	−0.11	0.10	0.93
*100% MCP*	−0.11	−0.53	0.48	0.91
*95% UD*	−0.79	−1.41	0.60	0.44
*50% UD*	1.26	−0.11	0.10	0.25
**Attendance Days Per Partner**				
*Sex* (*Male*)	−2.25	−7.42	−0.51	0.10
*Habitat* (*RH*)	0.77	−1.06	2.52	0.43
*SVL*	3.69	12.35	61.85	0.01
*MPD*	−0.08	−4.25	4.98	0.88
*DPM*	0.83	−2.29	8.96	0.26
*MMF*	−0.54	−1.51	1.16	0.80
*Directionality*	0.05	−0.94	0.89	0.96
*100% MCP*	−0.11	−5.84	2.51	0.45
*95% UD*	−1.04	−4.19	13.11	0.33
*50% UD*	1.11	−6.39	0.44	0.12

Reference levels for parameters are in parentheses (Male, RH = Resource Hotspot), and coefficients can be used to interpret the direction of individual effects.

### Movement and space use models

There was a significant main effect of sex on MPD (*F*_*1,27*_ = 10.08, *P* < 0.01), as males had greater MPD than females. Season also had a significant main affect (*F*_*1,32*_ = 12.12, *P* < 0.01), with MPD being larger during the mating season. There was no significant main effect of habitat category upon MPD for males or females (*F*_*1,34*_ = 1.70, P = 0.20). An interaction between sex and season (*F*_*1,32*_ = 5.29, *P* < 0.01) was illustrated by males increasing MPD during the mating season (*t*_43_ = 3.72, *P* < 0.01) while females exhibited no significant seasonal difference (*t*_31_ = 0.84, *P* = 0.41). The main effect of season on male MPD was primarily driven by NH males, as interval estimates from pairwise comparisons showed that NH males significantly elevated MPD during the mating season (t36 = 3.12, P < 0.01) while RH males had a smaller seasonal difference (t44 = 2.44, P = 0.05). The marginal and conditional pseudo-*R*^2^ measures of fit are 0.33 and 0.68, indicating a moderate level of fit.

There was a significant main effect of sex (*F*_*1,26*_ = 35.74, *P* < 0.01) on DPM, with males having greater DPM than females. Habitat also had a significant main effect (*F*_*1,33*_ = 12.14, *P* < 0.01), as DPM was significantly larger for NH males and females compared to RH males and females (*t*_47_ = 2.08, *P* = 0.04; *t*_32_ = 3.19, *P* < 0.01). Season did not have a significant main effect on DPM (*F*_*1,24*_ = 2.89, *P* = 0.10), but there was a significant interaction between season and habitat (*F*_*1,25*_ = 6.88, *P* < 0.01). Pairwise comparisons revealed that both NH males and females increased DPM during the mating season relative to the non-mating season (*t*_22_ = 2.32, *P* = 0.03; *t*_17_ = 3.07, *P* < 0.01) while RH males and females displayed no seasonal differences (*t*_34_ = 0.29, *P* = 0.77; *t*_19_ = −1.33, *P* = 0.20). The marginal and conditional pseudo-*R*^2^ measures are 0.56 and 0.89, indicating a good fit.

Sex did not significantly affect MMF (*F*_*1,14*_ = 1.86, *P* = 0.20), but there was a significant effect of season (*F*_*1,19*_ = 16.20, *P* < 0.01) that was largely driven by males substantially increasing movement frequency during the mating season (*t*_40_ = 3.55, *P* < 0.01). There was no significant main effect of habitat category on MMF (*F*_*1,20*_ = 1.96, *P* = 0.18), but males in RH displayed a marginally greater MMF than those in NH (*t*_45_ = −1.83, *P* = 0.07). There was a three-way interaction present between sex, season, and habitat (*F*_*1,21*_ = 4.12, *P* = 0.05), as males in NH, but not RH, moved more frequently during the mating season (*t*_35_ = 3.94, *P* < 0.01), while female MMF was not affected by season or habitat (*t*_29_ = 0.05, *P* = 0.96). The marginal and conditional pseudo-*R*^2^ measures are 0.30 and 0.50, indicating a somewhat poor level of fit.

Sex (*F*_*1,50*_ = 2.36, *P* = 0.13), season (*F*_*1,44*_ = 0.18, *P* = 0.56), and habitat category (*F*_*1,61*_ = 7.03, *P* = 0.15) had no significant effects on movement directionality. There was a significant interaction between sex and habitat (*F*_*1,55*_ = 5.14, *P* = 0.03). Pairwise comparisons showed that NH males made more linear movements than RH males (*t*_40_ = 2.53, *P* = 0.02), while there was no difference between NH and RH female directionality (*t*_21_ = 0.53, *P* = 0.59). The marginal and conditional pseudo-*R*^2^ measures of fit are 0.21 and 0.56, indicating a somewhat poor level of fit.

There was a significant main effect of sex on 100% MCPs (*F*_*1,27*_ = 34.33, *P* < 0.01), as males had larger home ranges than females (*t*_29_ = 5.75, P < 0.01). There was a significant main effect of season (*F*_*1,37*_ = 16.77, *P* < 0.01), as males and females had larger MCPs during the mating season relative to the non-mating season (*t*_36_ = 3.90, *P* < 0.01). Habitat also had a significant effect (*F*_*1,35*_ = 8.86, *P* < 0.01), with NH rattlesnake home ranges being larger than those for RH rattlesnakes. Pairwise comparisons showed that NH males had larger MCPs relative to RH males (*t*_50_ = 2.58, *P* < 0.01), while habitat had no significant effect on female MCPs (*t*_26_ = 1.50, *P* = 0.15). The marginal and conditional pseudo-*R*^2^ measures are 0.56 and 0.74, indicating a good fit.

There was a main effect of sex, as males had larger 95% UD home ranges than females (*F*_*1,21*_ = 28.64, *P* < 0.01). There was also a main effect of season (*F*_*1,6*_ = 7.77, *P* = 0.03), with 95% UDs being larger during the mating season. Habitat also affected 95% UDs (*F*_*1,30*_ = 12.82, *P* < 0.01), with NH home ranges being larger than RH home ranges. Pairwise comparisons show that males in NH increased 95% UDs during the mating season relative to the non-mating season (*t*_27_ = 2.67, *P* = 0.01), while RH males displayed no seasonal difference (*t*_38_ = 1.34, *P* = 0.19). NH females also had larger 95% UDs than RH females (*t*_19_ = 12.62, *P* = 0.02), but there were no seasonal differences in female 95% UDs (*t*_22_ = 1.10, *P* = 0.28). The marginal and conditional pseudo-*R*^2^ measures are 0.50 and 0.76, indicating a good fit.

For core use area (50% UD), there was a main effect of sex (*F*_*1,29*_ = 7.29, *P* = 0.01), as males had larger 50% UDs than females. Season also affected 50% UDs (*F*_*1,11*_ = 18.95, *P* < 0.01), as core use areas were larger during the mating season than the non-mating season. There was a main effect of habitat (*F*_*1,37*_ = 11.76, *P* < 0.01), as NH 50% UDs were larger than those for RH rattlesnakes. Pairwise comparisons show that core use areas generally followed the same seasonal pattern as 95% UDs (although not as strongly), where NH males increase core use area size during the mating season (*t*_12_ = 2.02, *P* = 0.05) and RH males display no seasonal difference (*t*_19_ = 1.65, *P* = 0.12). Habitat did not have a significant effect on female core use area (*t*_12_ = 1.50, *P* = 0.15). The marginal and conditional pseudo-*R*^2^ measures are 0.42 and 0.89, indicating a good fit.

### Reproductive behavior models

Observations of reproductive behavior yielded 36 unique male-female pairings, with 14 occurring in NH and 22 in RH. Males in NH were observed with (mean ± s.e.m.) 0.67 ± 0.24 females (range = 0–2) per mating season, compared to 1.29 ± 0.42 females (range = 0–3) per season for RH males. Females in NH were observed with 1.0 ± 0.38 males (range = 0–3) per mating season, compared to 1.63 ± 0.46 males (range = 0–4) per season for RH females. NH males attended females for 1.4 ± 0.27 days (range = 1–4) compared to 3.2 ± 0.97 days (range = 1–14) for RH males. There was a significant main effect detected for SVL upon attendance-days-per-partner (*t*_13_ = 2.93, *P* = 0.01), as both male and female SVL was positively related to number of days spent in attendance. SVL did not have a significant effect on the number of observed partners (*t*_14_ = 1.25, *P* = 0.17). Habitat category and all movement and space use metrics had no significant effect on reproductive behavior (Table 5). Sex of the radiotracked rattlesnake did not have a strong effect on the number of observed mating partners (i.e., males and females encountered a similar number of potential mating partners) or attendance-days-per-partner. The marginal and conditional pseudo-*R*^2^ measures of fit are 0.20 and 0.70 for the mating-partner model, indicating a moderate level of fit, and 0.51 and 0.64 for the female-attendance model, indicating a good fit.

## Discussion

For vertebrate mating systems without male parental care, males are expected to make the largest investment in mate-searching^9,55^. Among snakes, this is often displayed through a seasonal increase in male movement and space use^23^, but intrinsic sexual differences in spatial ecology have also been observed outside of mating periods^40^. We, therefore, expected male *C. atrox* to display greater movement and space use than females across both non-mating and mating seasons. Males were then expected to elevate movement and space use during the mating season relative to the non-mating season, while females display no seasonal shifts. We also predicted that males would make more linear movements compared to females and increase directionality during the mating season in efforts to efficiently locate reproductive females.

Male movement distances (MPD, DPM) and all space use estimates (100% MCP, 95% UD, 50% UD) are greater than female measures across seasons, but there are no differences between male and female MMF and Directionality. Males also exhibit the characteristic increase in all movement (*sans* Directionality) and space use measures during the mating season relative to the non-mating season. The lack of an increase in movement directionality by males during the mating season could indicate that targeted resources (i.e., prey, mating partners) are spatially dispersed similarly across seasons, but this is not supported by the seasonal differences observed for other spatial measures. Further, the relatively poor levels of fit inferred from the marginal and conditional pseudo-*R*^2^ measures for the MMF and Directionality models indicate that these variables are comparably less informative than the other movement and space use measures. Without considering habitat category, females display no seasonal differences in movement or space use measures.

Organisms can alter their behavior in response to environmental changes, and for searching individuals, habitat heterogeneity and the consequent spatial distribution of resources is fundamental to understanding both foraging^7^ and reproductive strategies^9^. The visibly greater vegetation density and surface water availability (Fig. 1) coupled with a higher density of prey for *C. atrox* (Fig. 2) support our designation of earthen tanks as resource hotspots relative to native habitats on IMRS. Given that movement and space use are linked to prey availability in vipers^56^, we expected NH and RH to have significant effects on the spatial patterns of proximate rattlesnakes. Specifically, we hypothesized that NH rattlesnakes would display greater movement and space use than RH rattlesnakes. Independent of behavioral season, mean annual measures generally support this prediction. NH males have substantially larger home ranges (100% MCP, 95% UD, 50% UD) than RH males, and NH females have significantly larger 95% UD home ranges than RH females. NH males and females also have significantly larger DPM than RH males and females. RH males also make less linear movements than NH males, possibly in efforts to remain within the relatively small earthen tank habitats (three tanks represented during this study covered 0.52, 1.04, and 1.20 Ha).

**Figure 4:**
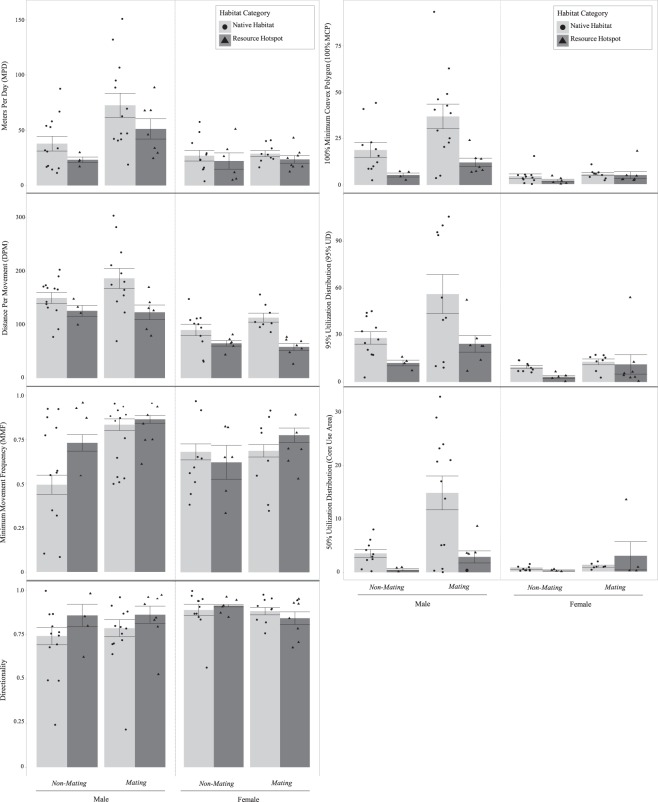
Mean (±s.e.m.) male and female *Crotalus atrox* movement and space use measures within behavioral season (Non-mating, Mating) and habitat category (Native Habitat, Resource Hotspot) and pooled across all sampling years (2015–2018). Movement measures (left) include MetersPerDay, DistancePerMovement, Minimum Movement Frequency, and Directionality. Space use estimators (right) include 100% Minimum Convex Polygon home range (100% MCP), 95% fixed-kernel Utilization Distribution home range (95% UD), and 50% fixed-kernel Utilization Distribution core use area (50% UD, core use area).

When accounting for season, the effects of habitat category become more apparent, and two distinct temporal patterns emerge (Fig. 4). NH males increase all movement (*sans* Directionality) and space use measures during the mating season relative to the non-mating season, while RH males display no seasonal differences characteristic of prolonged mate-searching behavior. The dichotomy between NH and RH male home range sizes is particularly notable, both in terms of the magnitude of the increase during the mating season by NH males and the relative difference between NH and RH males within seasons (Fig. 4). These divergent seasonal patterns by NH and RH males might reflect a relative difference in the cost-benefit trade-off associated with moving in each habitat type. As with other critical resources, if potential mating partners are spatially consolidated in the environment, reduced movement and space use are favored in mate-searching efforts^57,58^. If reproductive females are spatially clustered around RHs during the mating season (Fig. 3), this would explain our finding that the seasonal shift in motivational state (i.e., foraging vs. mating) does not accompany a concurrent shift in spatial strategy by RH males. Conversely, NH females display relatively little seasonal variation in dispersion, and NH males make longer and more frequent movements within larger home ranges during the mating season relative to the non-mating season. Unexpectedly, NH females increased DPM during the mating season, while RH females showed no seasonal shifts. Females can participate in mate location if the investment is low relative to the potential gain^9,10^. One hypothesis of female participation in snakes is the mate facilitation model, whereby females increase movement to improve detection by males via lipid-based pheromone signaling^59,60^. The seasonal increase in NH female DPM might reflect such “advertising movements.” However, increased movement can also reflect an increased need for food acquisition^56^, particularly in the case of females that undergo vitellogenesis following the mating season^31^. Unlike DPM, female home range sizes within habitat categories showed no seasonal differences (Fig. 4). Seasonal increases in movement within home ranges, but not home ranges overall, has been observed for other female snakes during mating periods^40,59^.

Specific male strategies tailored to female distribution have been previously reported for other rattlesnakes (*Crotalus*), including examples of prolonged searching without female-defense^18^, prolonged searching with female-defense^40^, and temporal shifts within a population between prolonged searching and female-defense^61^. Broadly, the mating system for *C. atrox* on IMRS can be characterized as intermediate between prolonged-search and female-defense polygynandry. We observed males and females mating with multiple partners within a single mating season, our spatial data show that some males make a substantial investment in mate-searching, and males occasionally exhibit extended female attendance. However, the divergent movement and space use patterns exhibited by NH and RH rattlesnakes might be indicative of two alternative strategies within the broader intermediate system. The substantial increase in movement and space use by NH males during the mating season and the brief attendance times observed (1.4 ± 0.27 days by NH males) both characterize prolonged mate-searching polygynandry. Although the reproductive behavior GLMMs show no strong relationships between NH male movement or space use and the number of observed mating partners, this result could be an artifact of our sampling protocols, given the relatively small sample of radiotracked males in NH that contributed reproductive data (N = 9) and that the number of mating partners for individual NH males ranges only from zero to two. A more compelling indication of the effectiveness of the NH male search strategy is that there’s no significant difference in the number of observed mating partners between NH (0.67 ± 0.24) and RH males (1.29 ± 0.42), despite their contrasting seasonal spatial patterns. The number of mating partners also do not differ significantly between NH (1.0 ± 0.38) and RH females (1.63 ± 0.46) (Table 5).

A narrow-sense mating system classified as “hotspot polygyny” can occur when females cluster within a small area due to a similar clustering of limiting ecological resources^12^. The index of dispersion (*I*) illustrates that radiotracked RH females exhibit a highly clustered distribution during the mating season, while all other groups show relatively little seasonal variation in *I* (Fig. 3). Spatial clustering of females in RH could potentially intensify male-male competition, and elevated female-defense behavior by males within a female-hotspot might be favored over prolonged-mate searching. In the case of RH males, this is partially corroborated by the lack of increases in movement or space use during the mating season and the lack of associations between RH male movement and the number of mating partners. Although there is not a significant habitat-specific difference in attendance-days-per-partner (Table 5), RH males did attend females for 3.2 ± 0.97 days compared to 1.4 ± 0.27 days by NH males. Alternatively, within a hotspot, multiple mating by females (observed on IMRS), multiple paternity of litters (reported elsewhere for *C. atrox*^17^), and a relative abundance of females, could potentially combine to narrow the typically male-biased operational sex ratio (OSR) and reduce the force of sexual selection on female-defense behavior^12^. The measures of dispersion (*I*) reported here, while derived from relatively small samples, serve as a preliminary indication of *C. atrox* spatial distribution patterns within NH and RH. True estimates of *C. atrox* densities within NH and RH would allow a detailed assessment of habitat-specific OSR, which is key to linking reproductive strategies to local conditions.

Strategic movement patterns have been widely studied in foraging organisms, but less frequently in the context of mate-searching behavior. This study system offered a unique opportunity to test how variation in local resource distribution might influence movement and space use across behavioral seasons by a pitviper. In general, sex and season-specific patterns reflect those expected under a male search-based polygynandrous mating system, as males move greater distances and use more space than females, and males substantially increase movement and space use during the mating season. However, by accounting for rattlesnakes using earthen tank habitats and those using native habitats, we document divergent seasonal search patterns relative to habitat category. NH rattlesnakes display greater movement and space use than RH rattlesnakes, and NH males increase movement and space use during the mating season while RH males show minimal (in the case of MPD) or no seasonal shifts. NH females also increase movement distance (DPM) during the mating season compared to no seasonal shift by RH females. RH males make significantly less linear movements than NH males, possibly indicating that RH males conform their movements patterns to remain within the small earthen tanks. Despite these contrasting spatial patterns, the number of observed mating partners and female-defense effort for NH and RH rattlesnakes does not differ. Our seasonal movement and space use results combined with observations of reproductive behavior might reflect alternative strategies by NH and RH *C. atrox* on IMRS. Additional sampling under a more frequent relocation schedule during the mating season combined with estimates of habitat-specific *C. atrox* densities could further elucidate the link between reproductive strategies and habitat categories in this system. Nonetheless, our study highlights the potential for multiple interacting mechanisms (i.e., sexual selection, habitat heterogeneity, behavioral plasticity) to facilitate divergent spatial strategies within populations and provides additional evidence for the dynamic role of ecological factors in the evolution of animal mating systems.

## Data Availability

The datasets generated and analyzed during this study along with the associated R code for analyses are available on GitHub: dldesantis/glmm_Movement-SpaceUse.
